# Prevention and Treatment of Bone Metastases in Breast Cancer

**DOI:** 10.3390/jcm2030151

**Published:** 2013-09-24

**Authors:** Ripamonti Carla, Trippa Fabio, Barone Gloria, Maranzano Ernesto

**Affiliations:** 1 Supportive Care in Cancer Unit, Department of Haematology and Pediatric Onco-Haematology Fondazione IRCCS, Istituto Nazionale dei Tumori di Milano, Milan 20133, Italy; E-Mails: carla.ripamonti@istitutotumori.mi.it (R.C.); gloria.barone@istitutotumori.mi.it (B.G.); 2 Oncology Department, Radiation Oncology Centre, Santa Maria Hospital, Via T. di Joannuccio, Terni 05100, Italy; E-Mail: f.trippa@aospterni.it

**Keywords:** breast cancer, bone metastases, radiotherapy, bisphosphonates, endocrine therapy, chemotherapy, bone pain relief

## Abstract

In breast cancer patients, bone is the most common site of metastases. Medical therapies are the basic therapy to prevent distant metastases and recurrence and to cure them. Radiotherapy has a primary role in pain relief, recalcification and stabilization of the bone, as well as the reduction of the risk of complications (e.g., bone fractures, spinal cord compression). Bisphosphonates, as potent inhibitors of osteoclastic-mediated bone resorption are a well-established, standard-of-care treatment option to reduce the frequency, severity and time of onset of the skeletal related events in breast cancer patients with bone metastases. Moreover bisphosphonates prevent cancer treatment-induced bone loss. Recent data shows the anti-tumor activity of bisphosphonates, in particular, in postmenopausal women and in older premenopausal women with hormone-sensitive disease treated with ovarian suppression. Pain is the most frequent symptom reported in patients with bone metastases, and its prevention and treatment must be considered at any stage of the disease. The prevention and treatment of bone metastases in breast cancer must consider an integrated multidisciplinary approach.

## 1. Introduction

Breast cancer is one of the most commonly diagnosed cancers among women in the industrialized world [1]. At diagnosis, approximately 5%–6% of women present with distant spread, and over 70% of these will have bone metastases (BM) during the course of disease [2].

Osteolytic, osteoblastic and mixed forms of metastasis are observed. The signs and symptoms of BM largely depend on the location and the mechanical stress on the affected parts of the bone. It manifests itself in pain, movement restrictions and skeletal-related events (SREs), which are usually defined as pathological fractures, spinal cord compression, bone pain requiring palliative radiotherapy (RT) and orthopedic surgery [3]. The first approach in the management of BM is the prevention of SREs, given their negative impact on quality of life and on worsening of the prognosis. Early diagnosis of BM is advisable with the aim of (1) changing the oncological therapies, (2) assessing and treating pain appropriately, (3) preventing SREs and (4) monitoring the patients over time. Although premature death is inevitable, remissions are frequent, and patients usually require supportive and palliative therapy for many months or years.

Treatment of BM and SREs often requires a multimodal approach (Table 1), considering medical, surgical, percutaneous procedures and RT, which plays a central role. Additional application of antiresorptive agents (e.g., bisphosphonates or the receptor activator of nuclear factor-kB ligand inhibitors) has proven successful [3,4].

**Table 1:** Treatment of Metastatic Bone Disease: Multidisciplinary approach.

Radiotherapy	The management of painful metastatic bone disease requires the use of multidisciplinary therapies such as hormone therapy or chemotherapy, external radiotherapy to the painful area or at time of risk of fracture or spinal cord compression, orthopaedic surgery, bisphosphonates, radionuclides and radiofrequency. Analgesics should be prescribed at any time.
Hormone therapy	
Chemotherapy	
Orthopedic surgery	
Analgesics	
Bisphosphonates	
Denosumab	
Interventional radiology (cementoplasty)	
Radionuclides	
Radiofrequency	

## 2. Clinical Presentation and Diagnostic Workup

**Figure 1:**
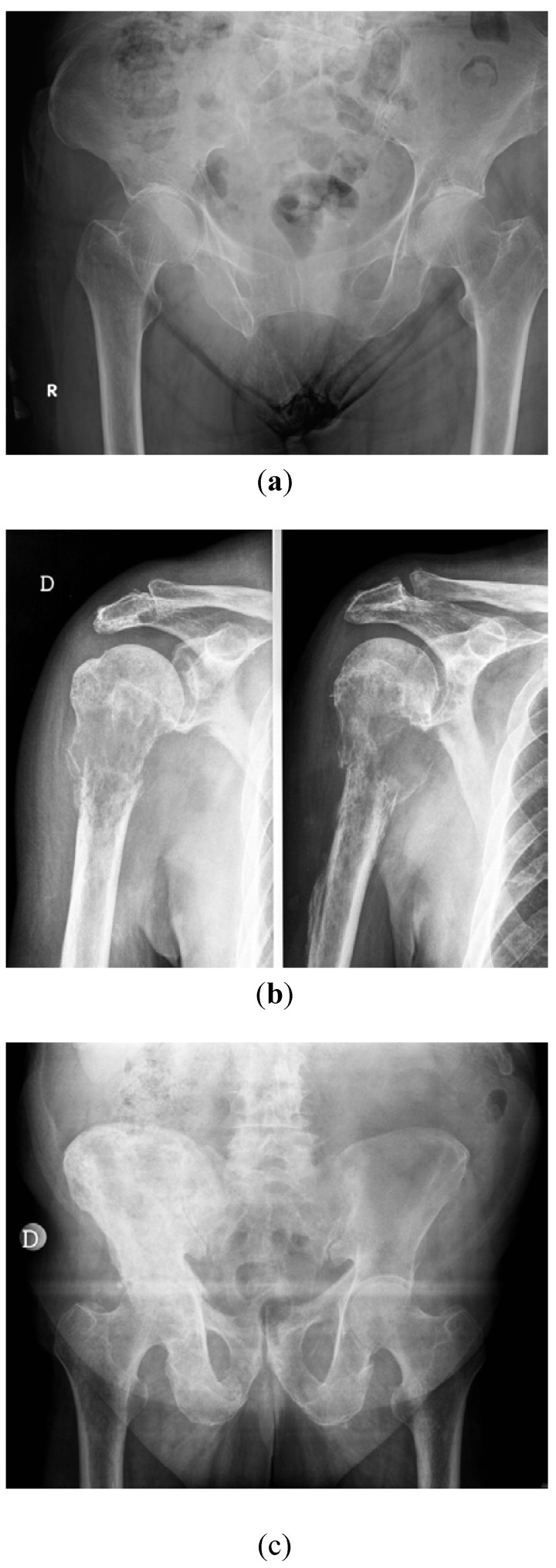
Standard X-ray examples of bone changes detectable in women with breast cancer: Osteoporosis of head femoral areas (**a**), pathological fracture of proximal third of the right humerus due to a large lytic lesion (**b**) and blastic bone infiltration of right hemipelvis (**c**).

The signs and symptoms of BM largely depend on the location and the mechanical stress on the affected parts of the bone. Pain is often registered as the first symptom of both osteolytic and osteoblastic BM and may be present in the absence of radiological evidence (standard X-ray) (Figure 1). In the presence of tumor infiltration, of compression of a nervous plexus or of pathological fracture with bone impingement, also, sensory and/or motor deficits can be registered. The degree of bone involvement or destruction, the amount of neural compression and the extent of systemic disease are all considered poor prognostic factors in breast cancer patients [3]. Adjuvant therapies for breast cancer increase the overall survival of patients with this disease, thus increasing the period during which bone lesions may arise. Therefore, in patients with breast cancer, the presence of pain and/or the other aforesaid symptoms cannot be under evaluated, because they can be suggestive of BM until proven otherwise. Bone scintigraphy is used for the diagnostic workup, allowing a total skeletal assessment, which is helpful for confirming the doubt of BM and the extent of the metastatic spread. Traditional X-ray imaging is used for the evaluation of suspiciously enhancing lesions. It permits a differentiation between lytic, blastic or mixed lytic-blastic BM. Computed tomography (CT) also shows smaller osteolysis, allows diagnosis of tumor extension into adjacent soft tissues and permits the evaluation of bone stability better than traditional X-rays. When a myelo-radicular compression is suspected or already diagnosed by CT, magnetic resonance imaging (MRI) has an important role in studying the local extension and the disease diffusion along all the spine.

## 3. Medical Strategies for Bone Metastases

The goals of the medical therapy after surgical intervention are the prevention of distant bone and visceral metastases. The adjuvant systemic treatment for breast cancer has the aim of reducing the risk of recurrence and death by means of poly-chemotherapy [5], endocrine therapy [5] and biological therapy (trastuzumab) [6,7,8,9,10,11,12,13,14], also through the prevention of BM.

Endocrine therapy is indicated in hormone receptor-positive tumors. Tamoxifen administered for five years is the treatment of choice for premenopausal or perimenopausal patients with resected hormone responsive breast cancer, regardless of other features of the tumor. Tamoxifen can also be administered in postmenopausal for 2–3 years, followed by the third-generation aromatase inhibitors (AI) for 3–2 years. In addition, postmenopausal tamoxifen is an alternative to the AI: For patients who refuse the AI and for patients for whom the use of AI is contraindicated. In women with tumors that are estrogen receptor positive, tamoxifen significantly reduces the annual risk of recurrence by 39% and death from breast cancer by 31%, compared to the control, independent of the use of chemotherapy, age, menopausal state, nodal status and the state of the progesterone receptor. At a median follow-up of 15 years, tamoxifen for five years results in a reduction of the absolute risk of recurrence and death of 11.8% and 9.2%, respectively [5].

Aromatase inhibitors are indicated in the adjuvant endocrine treatment of women with postmenopausal hormone responsive breast cancer. The option of treatment includes monotherapy for five years or the sequence for 3–2 years after tamoxifen for 2–3 years.

Two phase III randomized trials with an “upfront” strategy compared AI for five years (anastrozole in the Arimidex, Tamoxifen, Alone or in Combination (ATAC) trial, letrozole in the study, BIG 1-98) to tamoxifen for five years [15,16]. The ATAC trial showed an absolute advantage in disease-free survival (DFS) at a median follow-up of 100 months by 2.4%, but no significant benefit in overall survival (OS). The BIG 1-98 study showed an absolute advantage in DFS at a median follow-up of 76 months of 2.3% and a significant advantage in OS, but only for censored analysis, *i.e.*, excluding the patients (25, 2% of cases) that had the chance of changing the treatment from tamoxifen to letrozole after the first interim analysis, performed at a median follow-up of 25.8 months.

The data from the ATAC and BIG 1-98 were collected in a meta-analysis (9856 patients). Overall, it fixed an absolute advantage in DFS at a median follow-up of five and eight of 2.9% and 3.9%, respectively (*p* < 0.00001), with no advantage either in OS or in breast cancer mortality. The advantage in DFS was more evident in terms of reducing the risk of developing a contralateral breast cancer (HR = 0.59, *p* = 0.0009) and local recurrence (HR = 0.70, *p* = 0.003) and less obvious in terms of reducing the risk of distant recurrence (HR = 0.82, *p* = 0.002) [17].

To date, in adjuvant endocrine therapy in premenopausal patients, the role of luteinizing hormone releasing hormone (LHRH)-analogue in addition to tamoxifen or the combination of chemotherapy and tamoxifen should be considered uncertain. The addition of LHRH analogue to tamoxifen *versus* tamoxifen alone did not significantly reduce the risk of recurrence (HR = 0.85, *p* = 0.20) and death after recurrence (HR = 0.84, *p* = 0.33) [18]. Despite the addition of LHRH analogue to tamoxifen, it seems to have a marginal benefit in terms of recurrence and death; the use of such a combination prevents the increase in plasma levels of estradiol, which occurs with tamoxifen alone, reducing the toxicity that may result from ovarian cysts or metrorrhagia [19]. Combination chemotherapy is superior to single-agent chemotherapy. The regimens containing anthracyclines and taxanes are superior in DFS and in OS compared to regimens without taxanes.

The major randomized trials that compared regimens without taxanes to regimens with taxanes in the adjuvant treatment of patients with high risk of relapse (axillary nodes positive or negative) were included in the final meta-analysis, of the Early Breast Cancer Trialists’ Collaborative Group (EBCTCG) [20]. Thirty-three studies were taken into account that enrolled a total of 44,000 patients. Overall, the meta-analysis confirmed a reduction in the risk of relapse by 13% and the risk of death (from any cause) by 11% in favor of taxane-containing regimens. Restricting the analysis to studies in which the taxane (paclitaxel or docetaxel) was added concomitantly or in sequence to anthracyclines and compared to treatments that contain anthracyclines, benefits of taxanes of a similar entity were observed compared to that observed in the overall analysis. However, it was found, in contrast to previous meta-analyzes available [21], that there was a significant impact of anthracyclines doses without taxanes in comparison schemes. The benefit of the addition of taxanes to anthracyclines is maximal when the cumulative dose of anthracyclines in the two comparator arms is similar. In this case, the addition of the taxane determines a reduction of the risk of relapse and death by 16% and 14%, respectively, which results in a significant gain in PFS and OS to eight years by 4.6% and 3.2%, respectively. With an increasing dose of anthracyclines without taxanes in the comparator arm, the benefits in terms of both DFS and OS tend to decrease, canceling when the dose of anthracyclines in the comparator arm is double or more than that in the arm with taxanes.

The study by US Oncology [22] is the only one in the adjuvant setting that compared a regimen containing anthracycline (AC: doxorubicin 60 mg/m², cyclophosphamide 600 mg/m² every 21 days for four cycles) with a regimen containing taxanes, but without anthracyclines (TC: cyclophosphamide 600 mg/m^2^, docetaxel 75 mg/m² every 21 days for four cycles), showing a benefit in DFS and, in a five-year follow-up, even in OS [23]. Therefore, the TC scheme can be taken into account in patients with contraindications to the use of anthracyclines and CMF as an alternative to the scheme.

Trastuzumab, a monoclonal antibody for the extracellular domain of HER2, should be administered in patients with operated HER2-positive breast cancer. Globally, almost all studies of trastuzumab in the adjuvant setting have shown, excluding the studies with a smaller sample (PACS-04 and FINHER), a significant advantage in DFS and variable from 6% to 12.8% compared to the control, with administration for a year [23]. The advantage in OS was instead obtained only with the administration of trastuzumab in combination with chemotherapy (taxane), but not in sequence to it, with an absolute advantage variable from 3.2% to 5% at a mean follow up of 4.5 years [20].

The benefit of trastuzumab was evident at both local-regional and distant sites, at time to distant recurrence [24].

### 3.1. Bisphosphonates: Prevention of Skeletal Related Events, Bone Loss and Metastasis

Bisphosphonates (BPs) are a well-established, standard-of-care treatment option to reduce the frequency, severity and time of onset of the SREs in breast cancer patients with BM [2,25,26,27,28,29,30]. From many years, BPs, in particular zoledronic acid (ZOL), have been incorporated into clinical practice recommendations for these patients [31], and denosumab [32,33] has been approved in many countries for the delay of onset of SREs due to BM in breast or prostatic cancer patients.

The efficacy and safety of a reduced dosing frequency of ZOL in women with bone metastases due to breast cancer, treated previously with monthly zoledronic acid, was assessed in a phase 3 trial in 62 centers in Italy (ZOOM study) [34]. After one year of a monthly administration of zoledronic acid, for 425 patients, the patients were assigned to the 12-week group (209 patients) and 216 to the four-week group. The results showed that it was possible to maintain the therapeutic effects with the two different schedules of administration. However, median N-terminal telopeptide concentration changed from baseline more in the 12-week group than in the four-week group after 12 months (12.2% *vs*. 0.0%, *p* = 0.011) [34]. The effects on N-terminal telopeptide should be investigated further before changing practices, because in previous studies, high levels of N-terminal telopeptide (NTX) were associated with a poorer prognosis and a shorter time to first skeletal-related event and NTX levels decreased in response to treatment with zoledronate [35].

In patients treated with BPs or denosumab, preventive dental measures, after dental screening examination [36], are advocated to reduce the osteoncrosis of the jaw (ONJ) incidence [37], due to their efficacy in patients with BM, but not in oncological patients with osteoporosis, yet. Recent recommendations for ONJ include a conservative approach with intermittent prophylactic antibiotic therapy and rinses with oral chlorhexidine and debridement [38]; moreover, a careful sequestrum removal of necrotic bone is recommended [39,40].

Bone is the most common site for breast cancer metastases, and the bone microenvironment plays a crucial role in harboring disseminated tumor cells (DTCs). Therefore, agents that affect bone metabolism might not only prevent the development of bone lesions, but also provide meaningful reductions in the risk of relapse, both in bone and beyond [41].

Bone homeostasis is a function of osteoblastic and osteoblastic activity. Osteoblastic bone resorption is stimulated by receptor activator of nuclear factor kappa-B ligand (RANKL) and osteoprotegerin (OPG) (a RANKL decoy receptor, which regulates the availability of active RANKL). Estrogens and androgens are able to modify OPG secretion and, thus, to act on osteoblastic activity. Sex hormone levels decrease with age and post menopause. Consequently, OPG levels also decrease, and osteoclastic bone resorption increases, whereas bone mineral density (BMD) decreases, favoring osteopenia and osteoporosis. Moreover, secondary osteoporosis may occur as a result of cancer treatments that decrease sex hormone levels, including chemotherapies, AI, androgen depravation therapy and gonadotropin-releasing hormone (GnRH) agonists [42].

Bisphosphonates, through blocking malignant osteolysis and, consequently, reducing bone-derived growth factors and cytokines, prevent bone destruction and would be expected to render the “soil” less hospitable for the growth of the cancer “seed” within bone.

Thus, adjuvant breast cancer treatments can have longer-term deleterious effects on bone health. Data of the literature shows that BPs have a role in preventing and treating cancer treatment-induced bone loss (CTIBL). Adjuvant bone protection studies of zoledronic acid have clearly shown effective prevention of aromatase inhibitor-induced bone loss (AIBL) and have additionally provided evidence of an anti-tumor effect [43].

Various revisions of the literature have been published on adjuvant bone-targeted therapy to prevent metastasis in breast cancer patients [44,45].

The first studies in the adjuvant setting were carried out on oral clodronate to test the potential efficacy of bone-targeted agents in preventing metastasis in early stage breast cancer, with conflicting results [46,47,48].

Later, the Australian Breast and Colorectal Cancer Study Group (ABCSG)-12 trial enrolled 1803 premenopausal women to evaluate different endocrine strategies with and without six monthly zoledronic acid treatments on bone health and recurrence endpoints. All patients received ovarian suppression therapy with goserelin and were randomized to receive additional treatment with tamoxifen or anastrozole, either with or without ZOL. At a median time follow-up of 48 months, anticancer effects with ZOL were seen both in bone and beyond (less frequency of distant metastases, of locoregional relapses and contralateral breast cancer) [49]. More than three years after the completion of treatment, further analyses at 62 months [50] and at 84 months [51] showed a persisting benefit in disease-free survival (DFS). The recent analysis of ABCSG-12 suggested a statistically significant age effect on the impact of ZOL on both DFS and OS. In younger premenopausal patients, no statistically significant difference in DFS was observed between the ZOL group and the control group (*p* = 0.821). Differently among patients aged more than 40 years in whom complete ovarian suppression with goserelin was more likely, ZOL resulted in a 42% reduction (*p* = 0.013) in the risk of DFS events and a 43% reduction in the risk of death compared with the control group (*p* = 0.057) [51].

The trial “Does adjuvant zoledronic acid reduce recurrence in stage II/III breast cancer?” (AZURE) [52] includes 3360 breast cancer patients, who received chemotherapy and endocrine therapy and were randomly assigned to receive ZOL every 3–4 weeks for six doses and every 3–6 months, until five years or the first evidence of distant metastases. In the intention-to-treat population, the addition of ZOL did not significantly increase DFS compared with standard treatment alone at a median follow-up of 50 months. There was a trend toward an improvement in overall survival in patients treated with ZOL (*p* = 0.07). In postmenopausal women, in the ZOL arm, significant reduction of locoregional recurrence, new second primaries and non-skeletal distant recurrence was observed (*p* < 0.001).

ZO-FAST [53] is a European study designed to investigate the bone-preserving activity of zoledronic acid during adjuvant therapy with AI in postmenopausal women treated with letrozole and ZOL, 4 mg, every six months or delayed treatment with ZOL following a significant decrease in BMD (control group). After three years’ median follow-up, in addition to the BMD benefits with ZOL (primary endpoint), the upfront-ZOL group had a significant 41% reduction in the risk of DFS events *vs*. the delayed ZOL group (*p* = 0.0314).

### 3.2. Bone Pain: Assessment and Treatment during All the Illness Trajectories

The proper and regular self-reporting assessment of pain is the first step for an effective and individualized treatment. Pain is always a subjective sensation. Individualized pain management should take into account the onset, type, site, duration, intensity and temporal patterns of the pain (from this, it is often possible to define the cause of the pain), concurrent medical conditions and, above all, the subjective perception of the intensity of pain that is not proportional to the type or to the extension of the tissue damage, but that depends on the interaction of physical, cultural and emotional factors.

**Figure 2:**
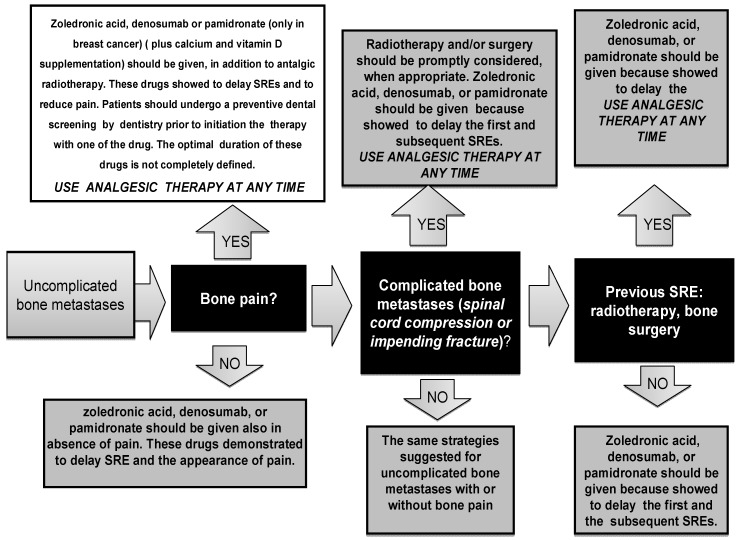
Treatment of pain due to bone metastases.

The proper and regular self-reporting assessment of pain (intensity and outcomes) with the help of validated assessment tools is the first step for an effective and individualized treatment. The most frequently used standardized scales are visual analogue scales (VAS), verbal rating scale (VRS) and the numerical rating scale (NRS). The assessment of all components of suffering, such as psychosocial distress, should be considered and evaluated [54]. Principles of pain management are described in Table 2. Figure 2 summarizes the treatment of pain in BM [54]. Analgesic therapy should be administered at any time of the clinical history of the patient and, also, before painful diagnostic procedures, such as bone biopsy.

**Table 2:** An effective pain-relieving therapy must considering the following issues.

1. Inform the patients about pain and pain management and encourage them to take an active role in their pain management.
2. Prevent the onset of pain by means of the “by the clock” administration, taking into account the half-life, bioavailability and duration of action of the different drugs; thus analgesics for chronic pain should be prescribed on a regular basis and not on “as required” schedule.
3. Prescribe a therapy which is simple to be administered and easy to be managed by the patient himself and his family, especially when the patient is cared for at home. The oral route appears to be the most suitable to meet this requirement, and, if well tolerated, it should be advocated as the 1st choice.
4. Prescribe rescue dose of medications (as required) other than the regular basal therapy episodic or breakthrough pain episodes.
5. Tailor the dosage, the type and the route of drugs administered according to each patient’s needs.

Uncomplicated bone metastases are lytic, blastic or mixed lesions without the risk of impending fracture or spinal cord compression.

Opioids are the mainstay of analgesic therapy and can be associated with non-opioid drugs, such as paracetamol or nonsteroidal anti-inflammatory drugs, and to adjuvant drugs (for neuropathic pain and symptom control). The role and the utility of weak opioids (*i.e.*, codeine, dihydrocodeine, tramadol) is a point of controversy. Morphine has been placed by the WHO [55,56] on its Essential Drug list. In a comparative study with other strong opioids (hydromorphone, oxycodone), there was no evidence to show the superiority or inferiority of morphine as the first choice opioid. Oral methadone is a useful and safe alternative to morphine. Methadone presents the potential to control pain that is difficult to control with other opioids. Although the oral route of opioid administration is considered the one of choice, intravenous, subcutaneous, rectal, transdermal, sublingual, intranasal and spinal routes must be used in particular situations. Transdermal opioids, such as fentanyl and buprenorphine, are best reserved for patients whose opioid requirements are stable. Switching from one opioid to another can improve analgesia and tolerability [54].

### 3.3. Pain at Rest and Pain on Movement

A distinctive feature of pain related to BM is that it may be absent or mild at rest, but may become severe or intolerable when exacerbated by different movements or positions (incident pain), such as standing, walking, sitting or turning. Moreover, pain induced by movement may be difficult to treat, even with rescue opioid medications, and is a predictor of poor pain control and leads to varying degrees of functional impairments and psychological distress.

Treatment of pain reported on movement in patients with BM is an important clinical problem, because it may be difficult to treat, even with rescue opioid medication, and creates functional impairment, as well as psychological distress.

Freedom from pain during movement is particularly difficult to achieve in patients with BM. An insufficient amount of opioids taken at regular intervals, as well as movement can cause acute episodes of pain that are predictable and not classifiable, known as unpredictable breakthrough pain (BTP). These BTP episodes are of moderate to severe intensity, with rapid onset (min) and a short duration (maximum 30 min) [57].

Breakthrough pain in cancer patients should be treated using a combination of pharmacological and nonpharmacologic interventions, including a careful optimization of background pain therapy to avoid the exacerbation of pain, due to the final dose analgesic effect, and the prescription of supplemental analgesic rescue therapy to prevent or treat the rapid onset of pain exacerbation.

Available pharmacological treatment options include oral, subcutaneous, intravenous transmucosal, buccal or nasal opioids; however, few RCTs are available [54].

On evaluating the role of BPs in achieving pain relief in patients with bone metastases, a Cochrane review [58] has identified thirty randomized controlled studies (21 blinded, four open and five active control), for a total of 3682 subjects included. For the proportion of patients with pain relief (eight studies) pooled data showed benefits for the treatment group, with an NNT at four weeks of 11 (95% CI 6–36) and at 12 weeks of seven (95% CI 5–12). In terms of adverse drug reactions, the NNT was 16 (95% CI 12–27) for discontinuation of therapy. Nausea and vomiting were reported in 24 studies, with a non-significant trend for a greater risk in the treatment group. The small number of studies in each subgroup with relevant data limited our ability to explore the most effective BPs and their relative effectiveness for different primary neoplasms. The overall conclusion from the Cochrane review was that there is enough evidence to support the effectiveness of BPs in providing some pain relief for bone metastases. There is insufficient evidence to recommend BPs for an immediate effect, as a first line therapy. Bisphosphonates and denosumab should be considered for the prevention of SREs, also, where analgesics and/or RT are adequate for the management of painful BM [54,59].

## 4. Radiotherapy

The goals of palliative RT are pain relief, recalcification and stabilization of the bone, as well as reduction of the risk of complications (e.g., bone fractures, spinal cord compression). The exact time of pain relief derived from RT is actually not known; generally, it is observed within a few days after the start of RT. Instead, radiologically detectable recalcification and stabilization are to be expected at the earliest within 6–12 weeks after termination of RT [60,61].

In case of singular or oligo-BM, high-dose RT can stop disease progression with the expected complications of the affected skeletal manifestation (e.g., pathological fracture and spinal cord compression) [2].

### 4.1. Radiotherapy for Bone Pain Relief

Radiotherapy has specific and critical efficacy in providing pain relief caused by cancer; however, the effectiveness depends largely on the radiosensitivity of the tumor, and in clinical practice, a difference of radiosensitivity has not been clearly integrated in therapeutic strategy (Figure 2). Painful BM are the most common indication for the use of palliative RT, with numerous prospective trials over the past three decades documenting improvements in pain from 60% to 80% after treatment, and in clinical practice, different fractionation regimens are used [62,63]. Recently, the American Society for Radiation Oncology, reviewing randomized published data regarding the use of RT in patients with BM, showed a pain relief equivalency for different regimens, including 30 Gy in 10 fractions, 24 Gy in six fractions, 20 Gy in five fractions and a single 8-Gy fraction [64,65].

Fractionated schedules of RT have been associated with an 8% repeat treatment rate to the same anatomic site because of recurrent pain *vs*. 20% after a single fraction. However, the long-term results concerning pain relief after re-irradiation were thus comparable with a palliative effect of more than 70% [66,67,68].

In clinical practice, the single dose approach optimizes patient and caregiver convenience. Therefore, a hypofractionated schedule can be considered the regimen of choice in patients with BM [69]. Furthermore, repeat irradiation to the same site of bone disease might be safe, effective and less necessary in patients with poor prognosis [70].

Recently, the breast cancer expert panel of the German Society for Radiation Oncology has developed guidelines for palliative RT. In their conclusions, the authors confirm that the appropriate fractionation should be chosen with respect to the treatment duration, estimated life expectancy and need for hospitalization [71]. Generally, patients in a good general health status would rather select fractionated irradiation. Therefore, the decision should be taken with the agreement of the radiation oncologist and patient. In conclusion, for patients with low performance status and pain, complex irradiation techniques and multi-fraction are, in general, not of increased value, due to prolonged treatment times, as well as the higher needs of precise positioning and immobilization. Simple techniques with a single dose of RT (*i.e.*, 8 Gy) should be used when analgesia is the main goal of treatment.

### 4.2. Radiotherapy for Bone Recalcification

Fractionated RT regimens (30 Gy in 10 or 20 Gy in five fractions, respectively) can be associated with better results concerning bone recalcification [61,72].

Generally, recalcification and stabilization of bone lesions are detectable with X-ray from 6 to 12 weeks after RT. This result is ameliorated by concomitant administration of bisphosphonate, while simultaneous application of denosumab are still outstanding [73]. In the case of expanded BM, with high risk of fractures, stabilization with surgery must be evaluated before RT.

### 4.3. Radiotherapy for Metastatic Spinal Cord Compression

Bone metastases are defined as complicated in the case of impending fracture or spinal cord compression.

A patient with an impending fracture (*i.e.*, a large lytic metastasis, which can be the cause of pathological fracture) must be evaluated for a surgery stabilization procedure before RT. If the surgeon does not give a surgical indication, RT alone represents the treatment of choice to control local disease and pain (Figure 2) [54].

Metastatic spinal cord compression (MSCC) can be induced by tumor infiltration of the spinal space or by intraspinal metastases. Spinal cord compression is one of the most dreaded complications of metastatic cancer, occurring in 5%–10% of all cancer patients during the course of their disease and requiring urgent oncologic care. Autopsy studies suggest that approximately one third of patients with solid tumors may have metastases to the spine, but the clinical evidence of MSCC is estimated in 3% to 7% of patients [74,75]. Approximately 50% of MSCC cases in adults arise from breast, lung or prostate cancer, but MSCC has also been described in patients with lymphoma, melanoma, renal cell carcinoma, thyroid carcinoma, sarcoma and myeloma. Pain accompanies spinal cord compression in approximately 95% of adults and 80% of children with MSCC and usually precedes the diagnosis by days to months. Weakness, the second most common symptom at presentation, usually follows the development of local or radicular pain and generally progresses to plegia over a period of hours to days. Other symptoms of MSCC are sensory loss and incontinence, which typically develop after the pain [76].

On suspicion of MSCC, magnetic resonance imaging should be performed. Magnetic resonance imaging has a sensitivity of 93%, a specificity of 97% and an overall diagnostic accuracy of 95% [74].

Prognosis is, above all, related to early diagnosis and therapy. The speed of neurologic deficit onset can condition the functional outcome, which is significantly better with slower development of motor dysfunction before RT. One study evidenced that ambulatory recovery occurred in 86% and 35% of patients with a history of >14 days compared with one to seven days, respectively [77]. Survival after MSCC is related to primary tumor type, ranging from 17–20 months for breast, prostate and myeloma to only four months for lung. If untreated, the majority of patients with MSCC becomes paraplegic. Early detection and treatment when the patient is still able to walk results in the highest chance of ambulation.

In MSCC, the aim of treatment is to improve the patients’ quality of life through control of back pain and preservation or recovery of motor and sphincter functions. Although it could be questionable if local treatment increases patients’ survival, there is a tight relationship between survival time and functional status. In fact, MSCC patients who had no motor dysfunctions live longer than paraparetic and paraplegic ones and, generally, die of systemic tumors, rather than local progression at the spine [78]. Considering that treatment success is related to the severity of the epidural disease and to the patient clinical condition at the time of diagnosis, it is important to perform diagnosis early and to begin treatment before significant myelopathy develops.

In clinical practice, MSCC can be treated with surgery followed by RT or RT alone, and the choice of treatment depends on patient selection. Once the diagnosis of MSCC is made, steroids are generally prescribed to control edema and to lessen pain.

Surgery plays an important role in selected cases. On the basis of literature evidence, it can be concluded that initial surgical resection followed by RT should be considered for a carefully selected group of patients that has with single-level MSCC and neurological deficits. Other possible indications for surgery include the necessity of stabilization, vertebral body collapse causing bone impingement on the cord or nerve root, compression recurring after RT and an unknown primary requiring histological confirmation for diagnosis [75]. However, when there are diagnostic doubts, tomography-computed guided percutaneous vertebral biopsy can be an alternative to open surgery to avoid surgical side effects and to reduce incisional pain and the recovery period.

Although RT is an effective approach for the majority of MSCC patients, the optimal radiation schedule remains unknown. Except for some particular circumstances, the use of conventional fractionated RT (2 Gy per day to a total dose of 30–50 Gy in 3–5 weeks) has been abandoned in favor of RT regimens requiring a smaller number of fractions. Since 2005, there have been published two phase III randomized multicenter Italian trials [79,80]. The first compared a short-course regimen (*i.e.*, 8 Gy repeated after one week to a total dose of 16 Gy) to a split-course regimen (*i.e.*, 5 Gy × 3, four days rest and then 3 Gy × 5) [79]. The second compared the same short-course regimen to 8 Gy in a single fraction [80]. It is worth noting that both of these studies were performed in patients with short life expectancy (≤6 months) and that responders maintained function until death. While both hypofractionated RT regimens adopted has effective results, the authors concluded that the 8-Gy single fraction is the best option, considering that it is well tolerated, effective and convenient in this setting of patients. Published retrospective and prospective non-randomized data support the above randomized data in that no dose fractionation schedule has demonstrated with a higher ambulation rate [81]. However, some experience suggested that in MSCC patients, the duration of local control is superior, and consequently, the rate of in-field recurrences is lower following long-course RT regimens; these data add further weight to the argument for selecting a patient’s treatment based on prognosis [82]. Recently, it published a score predicting post-RT ambulatory status [83]. It was developed based on 2096 retrospectively evaluated MSCC patients and considered six prognostic factors (*i.e.*, tumor type, interval between tumor diagnosis and MSCC, presence of other bone or visceral metastases at the time of RT, pre-treatment ambulatory status and duration of motor deficits). This scoring system has been validated prospectively for the endpoint survival and ambulatory function. In conclusion, evidence suggests that until further randomized data is available, short-course/single fraction regimens (e.g., 5 × 4 Gy or 1 × 8 Gy) can be used for patients with short life expectancy, while fractionated, higher dose schedules (e.g., 10 × 3 Gy or greater) should be considered for patients with better prognosis.

#### Steroids for Metastatic Spinal Cord Compression

Generally, in MSCC patients, RT is administered with concomitant steroids to lessen back pain, prevent progressive neurologic symptoms and reduce radiation-induced spinal edema [84]. Steroids should be given immediately when the clinical-radiologic diagnosis of MSCC is obtained. Dexamethasone is the most frequently used drug, although the use of methylprednisolone is also reported. The dexamethasone dose ranges from moderate (16 mg/die in two four-times daily parenteral or oral divided doses) to high (36–96 mg/die), eventually preceded by a bolus of 10–100 mg intravenously [74]. The steroids are usually tapered over two weeks. No study has been published comparing high dose to moderate dexamethasone dose. There is only one randomized clinical trial comparing high-dose dexamethasone to no drug in 57 patients with MSCC treated with RT. This trial evidenced that high dose dexamethasone significantly improves post treatment ambulation, but is accompanied by a certain probability (11%) of high toxicity [84]. A phase II trial showed the feasibility of treating patients with MSCC, no neurologic deficits, or only radiculopathy, and no massive invasion of the spine at MRI or CT with RT (3 Gy × 10) without steroids [85]. However, in clinical practice, considering that published studies have shown no difference in outcome between high-dose and moderate-dose dexamethasone and the relatively high incidence of side effects from steroids, above all, in patients with diabetes mellitus, hypertension and peptic ulcer, a moderate dexamethasone dose (e.g., 16 mg/die two-times-daily) is suggested for symptomatic MSCC patients.

For treatment of MSCC, chemo-hormonal therapy can be used in combination with RT or alone in adults who are not surgical or radiation candidates, but who have chemo-hormonal sensitive tumors, such as lymphoma, small cell lung carcinoma, myeloma, breast, prostate or germ cell tumors. In children, chemotherapy is the primary treatment for chemo-responsive tumors.

### 4.4. Radioisotope for Bone Metastases

Radioisotope treatment can be considered an interesting palliative care option for pain control in patients with generalized BM. Moreover, the published data do not suggest that radioisotope therapy alone can substitute RT in these cases. However, radioisotope treatment is limited by the kind of BM, which must be osteoblastic, documented by a technetium-99 bone scan. A sufficient interval should be kept after a previous myelotoxic chemotherapy or half body irradiation (4–6 weeks). In selected cases, retreatment with radioisotope can be proposed, but should only be performed after the regeneration of blood cell count. This also applies to a planned chemotherapy or RT after radioisotope, since myelosuppression can occur with some delay.

A small number of trials have shown that radioisotopes can relieve bone pain in patients with breast cancer and lung cancer, while inconsistent results were produced in patients with hormone-refractory prostate cancer [86,87,88].

Particularly, the therapeutic efficacy of radioisotopes (*i.e.*, strontium-89-chloride and 186Re-1,1-hydroxyethylidene bisphosphonate) was evaluated in the palliation of painful BM in 50 patients with breast cancer. The global response rate ranged from 84% to 92%, with a median duration of pain relief of four months (range, 2–14); treatment was safe and feasible, with a moderate hematological toxicity [88].

Moreover, a recent systematic review evidenced a small benefit of radioisotopes for complete or partial relief in the short and medium term (1–6 months), with no modification of the analgesics used [89]. Therefore, the use of radioisotope treatment can be appropriate in circumstances in which patients have several sites of painful osteoblastic metastases in an anatomic area greater than that which could be safely treated with external RT.

### 4.5. New Radiotherapy Technologies for Bone Metastases

Linear accelerator technology has evolved with multileaf collimation, intensity modulated irradiation, systems of image guidance and robotic technology. These new technologies permit one to administer to a target volume a dose escalation of RT by ensuring radioablative dose delivery to tumor, while, at the same time, avoiding an excessive dose to surrounding critical normal tissue organs. Stereotactic body RT has emerged as a new treatment option in the multidisciplinary management of BM, particularly for lesions located within or adjacent (paraspinal) to vertebrae or the spinal cord. Radiation-induced myelopathy has a relevant late toxicity, because it may result in severe neurologic dysfunction. Higher RT doses, larger doses per fraction and previous exposure to radiation could be associated with a higher probability of developing radiation-induced myelopathy [74].

Therefore, stereotactic body RT provides an attractive option to deliver high-dose per fraction radiation, typically in a single dose (*i.e.*, 10–16 Gy) or in hypofractionation (*i.e.*, 9 Gy × 3 fractions or 6 Gy × 5 fractions) [90,91,92]. Moreover, stereotactic body RT was used in selected retrospective single-institution reports, and the endpoints of these studies were also heterogeneous. A radiation therapy oncology group is carrying on a phase II/III study (Radiotherapy Oncology Group—RTOG protocol 0631) of image-guided stereotactic RT *versus* conformal RT for localized spine metastasis. Considering the little available data on stereotactic RT for spine metastasis and awaiting the results of the aforesaid RTOG study, the American Society for Radiotherapy and Oncology-ASTRO guidelines suggest the use of this new technology only in selected cases (e.g., re-irradiation of spinal metastasis) [64].

## 5. Surgical Approaches for Bone Metastases and Metastatic Spinal Cord Compression

The goals of surgical intervention in patients with BM are to reduce pain and to improve function if no surgical treatment fails. Treatments for painful BM may not only diminish pain, but also may improve quality of life and independence/mobility and reduce skeletal morbidity, potential pathologic fractures, MSCC and other SREs.

The surgical management of established BM must be individually tailored to each patient. Multidisciplinary support and communication with regard to a patient’s performance status and response to oncological treatments will assist the orthopedic surgeon in identifying the surgical intervention. Surgical implantation must not only allow immediate stability for mobilization, but also provide a reliable, durable and long-term construct that matches the expected long-term outcome of the patient. The decision to proceed with surgery is based on a variety of factors, including severity of symptoms, location of tumor, expected morbidity if a fracture were to occur, expectations of the patient and viability of alternative or adjuvant treatments. It is worth noting that the patient must be fit for surgery, and the recovery and rehabilitation phase should not exceed the patient’s life expectancy. Moreover, the surgical construct should be sufficiently durable to last throughout the patient’s lifetime. This can be difficult to estimate and is based on multiple factors, such as age, comorbidities and the extent of visceral and skeletal disease. Even if the patient is expected to survive for >3 months, the pain relief from stabilization of a fractured humerus, femur or tibia is substantial. Asymptomatic lesions need only be followed clinically and radiographically. These lesions can be effectively managed with medical treatment, including RT, bisphosphonate therapy and treatment of primary tumor [93].

In clinical practice, patients treated with surgery for BM are routinely submitted to postoperative RT. Although there are quite limited published data describing the outcomes among these patients, postoperative RT was generally associated with patients regaining normal use of their extremity (with or without pain) and undergoing fewer reoperations to the same site [94].

In patients with metastatic spine tumor, the goals of surgery include pain relief, restoration and preservation of neurologic function and stabilization of the vertebral column. In appropriately selected patients, surgery has provided a significant improvement of quality of life, pain control, functional status and the ability to undergo adjuvant therapy. Minimally-invasive, or minimal-access, spine surgery is also evolving in the management of patients with spinal metastases and epidural disease not causing cord compression. The aim is to minimize the surgical morbidity, while maintaining efficacy by still performing a radical tumor resection, stabilizing the spine with implants and/or injecting cement via endoscopic technology, which often can be performed as outpatient. Kyphoplasty and vertebroplasty have been used in the treatment of vertebral compression fractures for patients with spine metastases. Both techniques involve the injection of chemical cement (polymethyl methacrylate) into the vertebral body under image guidance. Kyphoplasty involves balloon insertion first into the vertebral body, to create a cavity and to augment the vertebral body, and low pressure cement injection. Vertebroplasty involves high pressure injection of cement into the vertebrae without cavity creation. A randomized trial is ongoing to determine the appropriate use of these procedures. These techniques have been reported to provide immediate and sustainable pain relief from malignant spine compression fracture [95,96]. The up-front spinal stabilization and rapid pain relief is potentially advantageous for subsequent RT, given that patients require strict immobilization and co-operation for RT. A preliminary efficacy and safety for the combined treatment of kyphoplasty and stereotactic body RT has been reported [97]. A novel combination approach was recently reported with samarium-153-ethylene diamine tetramethylene phosphonate mixed with bone cement and injected using kyphoplasty into painful vertebral metastases [98]. Although only the preliminary feasibility can be commented on, this represents an innovative approach to combine stabilization with direct deposition of radiation into a diseased, fractured vertebrae.

In patients with MSCC, posterior decompressive laminectomy removes the lamina and posterior spinous process with no attempt to remove the anterior vertebral body tumor. This surgical approach has the disadvantage of not removing the tumor effectively, often resulting in inadequate decompression and the potential of worsening spine stability [99,100,101]. Decompressive laminectomy is usually used urgently to restore the neurological status in patients with widespread metastasis and is reserved as a salvage therapy [102]. Direct circumferential decompressive surgery came on board with advances in surgery and stabilization instrumentation. It is aimed at removing the anterior vertebral body tumor and decompressing the spinal cord. It is usually performed from both anterior and posterior approaches with anterior column reconstruction to stabilize the spine. This surgical approach, together with RT, has been shown to improve the ambulatory function in selected group patients with favorable prognosis. However, this is a major operation with the potential for prolonged hospitalization and rehabilitation.

## 6. Conclusions

Bone metastases are a common complication of breast cancer, and proper treatment is crucial, considering the long-term survival generally associated with patients affected by this condition. The prevention of SREs represents an important goal to avoid the severe symptoms often related to complications.

Various medical approaches are available for this setting of patients. Radiotherapy is a useful treatment option that can effectively control pain and prevent neurological dysfunction. Surgery is a valid tool that has to be considered in selected cases.

Early diagnosis and appropriate therapy represent the major goals that can be better achieved by a multidisciplinary team.
