# Correction: Ripamonti, C.; Trippa, F.; Barone, G.; Maranzano, E. Prevention and Treatment of Bone Metastases in Breast Cancer. *J. Clin. Med.* 2013, *2*, 151–175

**DOI:** 10.3390/jcm4121955

**Published:** 2015-12-10

**Authors:** Carla Ripamonti, Fabio Trippa, Gloria Barone, Ernesto Maranzano

**Affiliations:** 1 Supportive Care in Cancer Unit, Department of Haematology and Pediatric Onco-HaematologyFondazione IRCCS, Istituto Nazionale dei Tumori di Milano, Milan 20133, Italy; carla.ripamonti@istitutotumori.mi.it (C.R.); gloria.barone@istitutotumori.mi.it (G.B.); 2 Oncology Department, Radiation Oncology Centre, Santa Maria Hospital, Via T. di Joannuccio, Terni 05100, Italy; f.trippa@aospterni.it

The authors wish to make the following corrections to this paper [1]:

The authors’ names:

“Ripamonti Carla” should be “Carla Ripamonti”;

“Trippa Fabio” should be “Fabio Trippa”;

“Barone Gloria” should be “Gloria Barone”;

“Maranzano Ernesto” should be “Ernesto Maranzano”;

The authors would like to apologize for any inconvenience caused to the readers by these changes.
